# Trends and seasonal variation in the incidence and prevalence of irritable bowel syndrome in Korea: a multicenter OMOP CDM study

**DOI:** 10.3389/fpubh.2026.1711919

**Published:** 2026-06-05

**Authors:** Seung-hyo Hong, Nahyun Jeong, Jae-Woo Park, Jinsung Kim, Seok-Jae Ko

**Affiliations:** 1 Department of Clinical Korean Medicine, Graduate School, Kyung Hee University, Seoul, Republic of Korea; 2 Division of Digestive Diseases, Department of Korean Internal Medicine, Kyung Hee University Korean Medicine Hospital, Seoul, Republic of Korea; 3 Department of Digestive Diseases, College of Korean Medicine, Kyung Hee University, Seoul, Republic of Korea; 4 Department of Korean Internal Medicine, Kyung Hee University College of Korean Medicine, Kyung Hee University Hospital at Gangdong, Seoul, Republic of Korea

**Keywords:** environmental factors, epidemiology, irritable bowel syndrome, OMOP Common Data Model, prevalence and incidence, Rome IV criteria, seasonality

## Abstract

**Background:**

Irritable bowel syndrome (IBS) is a prevalent functional gastrointestinal disorder. Despite revisions of diagnostic criteria, studies applying the Rome IV criteria in Korea are limited. Environmental factors such as climate and season have been suggested to influence the epidemiology of IBS; however, their associations remain unexplored.

**Methods:**

We analyzed the prevalence and incidence of IBS in Korea from 2019 to 2023 using clinical data from eight nationwide hospitals converted to the Observational Medical Outcomes Partnership (OMOP) Common Data Model (CDM). Stratified analyses were conducted by year, month, sex, and age. Seasonal variations were examined in relation to meteorological factors, including temperature and humidity.

**Results:**

Both the prevalence and incidence of IBS decreased during the study period. Females consistently showed higher rates than males, although the sex gap in incidence narrowed over time. The highest incidence rates were observed in individuals aged ≥65 years and those aged 15–19 years. Seasonal variation was evident, with peak incidence in autumn, followed by summer, significantly correlating with humidity and temperature.

**Conclusion:**

This multicenter study highlights sex- and age-specific differences in IBS epidemiology in Korea and demonstrates significant seasonal associations with meteorological factors. These findings underscore the need for large-scale, prospective surveys and public health strategies to address the IBS burden.

## 1 Introduction

Irritable bowel syndrome (IBS) is a prevalent functional gastrointestinal disorder characterized by recurrent abdominal pain and altered bowel habits (1). While the global prevalence of IBS is estimated at approximately 11.2% (2), the introduction of the more stringent Rome IV criteria in 2016 has significantly altered epidemiological estimates worldwide (3, 4). In Korea, IBS remains a major healthcare burden, frequently prompting medical visits (5, 6). However, there is a scarcity of large-scale, population-based studies applying the recent Rome IV criteria to assess the updated epidemiology of IBS in Korea.

Furthermore, geographical and temporal variations in IBS incidence suggest a significant role for environmental factors, yet seasonal trends remain underexplored (7). The rationale for investigating seasonality in IBS lies in the multifactorial pathogenesis of the disease. Seasonal changes in temperature and humidity can alter gut microbiota composition, potentially influencing visceral hypersensitivity and intestinal permeability (8). Additionally, seasonal peaks in viral or bacterial gastrointestinal infections can act as a primary trigger for post-infectious IBS (PI-IBS) (9). Because Korea experiences four distinct seasons with significant variations in meteorological factors (10), it provides an ideal setting to investigate the impact of seasonality and climate on IBS onset. Despite this, empirical studies linking IBS incidence with objective meteorological data are currently lacking.

To address these gaps, we utilized the Observational Medical Outcomes Partnership (OMOP) Common Data Model (CDM), which standardizes diverse electronic health records across multiple institutions into a common format, thereby enabling robust, large-scale multicenter analyses (11). Using OMOP-CDM data from eight nationwide hospitals in Korea between 2019 and 2023, this study aimed to: (i) estimate the recent prevalence and incidence of IBS stratified by year, month, sex, and age; and (ii) evaluate the association between IBS incidence and seasonal/meteorological factors. Ultimately, we sought to provide updated epidemiological insights to inform targeted healthcare strategies and future directions for IBS management.

## 2 Methods

### 2.1 Data source

We analyzed medical care utilization for IBS and the prevalence and incidence of IBS in Korea using claims data from the CDM.

The CDM data were extracted from eight available institutions with data for IBS from 2019 to 2023, linked with Feedernet, which is a big data healthcare platform that converts electronic medical record data from domestic healthcare institutions into OMOP-CDM data and manages the data (11). The eight institutions were affiliated with Research Border Free Zone of the Korea CDM data network, which recognizes the Institutional Review Board (IRB) approval of the research organizing center and waives the need for individual IRB approval. The eight institutions were Ajou University Hospital, Bucheon Sejong Hospital, Daegu Catholic University Medical Center, Ewha Woman's University Medical Center, Kangdong Sacred Heart Hospital, Kangwon National University Hospital, Korea Cancer Center Hospital, and Kyung Hee University Hospital. All methods were carried out in accordance with relevant guidelines and regulations. The study protocol was reviewed and determined to be exempt from review by the IRB of Kyung Hee University Hospital at Gangdong (IRB No. 2024-10-009-001). The requirement for informed consent was waived because this study involved the retrospective analysis of de-identified data derived from the CDM, which posed no more than minimal risk to the subjects and made obtaining individual consent impracticable.

### 2.2 Research subjects

This study was performed using CDM data for the Republic of Korea from January 2019 to December 2023. The Korean Classification of Diseases, seventh revision (KCD-7), a diagnostic coding system analogous to the International Classification of Diseases, Tenth Revision, was used to categorize the diagnoses in the CDM. We included data associated with a primary diagnosis of IBS and with the following sub-diagnosis KCD-7 codes related to IBS: K580 (irritable bowel syndrome with diarrhea), K581 [irritable bowel syndrome with predominant diarrhea (IBS-D)], K582 [irritable bowel syndrome with predominant constipation (IBS-C)], K583 [irritable bowel syndrome with mixed bowel habits (IBS-M)], K588 (other and unspecified irritable bowel syndrome), and K589 (irritable bowel syndrome without diarrhea).

### 2.3 Definition of incidence and prevalence

To address the specificity of hospital-based electronic health records, the denominators for all estimates were defined as the total number of unique patients who had at least one medical encounter (outpatient or inpatient) during the specific observation period (year or month).

Prevalence was calculated as the proportion of patients with a recorded IBS diagnosis among the total hospital-visiting population within a given timeframe.

Incidence was defined as the number of patients receiving a first-time IBS diagnosis during the study period. To distinguish incident cases from prevalent ones, we applied a washout period of 1 year (365 days) prior to the index date; patients with any IBS diagnosis record in the 365 days prior to the index date were excluded from the incidence denominator.

### 2.4 Meteorological data linkage

Daily meteorological data, including mean ambient temperature (°C) and relative humidity (%), were obtained from the open portal of the Korea Meteorological Administration (KMA) (10). Given the relatively compact geographic area of the Republic of Korea and the uniform macroscopic seasonal changes across the country, we utilized nationwide average meteorological data rather than hospital-specific local data. The daily nationwide data were then aggregated into seasonal and monthly means to align with the clinical data for analyzing IBS incidence and prevalence.

### 2.5 Statistical analysis

Continuous variables are presented as means with standard deviations, and categorical variables as frequencies and percentages. To evaluate the association between environmental factors and IBS incidence, we utilized multivariable linear regression models. The regression analyses were conducted using aggregated year-season-level data, with the seasonal IBS incidence rate per 100,000 persons as the dependent variable. Mean seasonal relative humidity and mean seasonal temperature were evaluated as the primary meteorological predictors in separate models. Univariable models included each meteorological factor alone, whereas multivariable models additionally included calendar year as a categorical covariate to account for secular trends during the study period. Because the regression models were based on aggregated seasonal incidence data, individual-level covariates such as sex and age were not included in these meteorological models; sex- and age-specific incidence patterns were analyzed separately. To explore potential delayed effects, we also tested 1-season lag effects by associating meteorological data from the preceding season with current seasonal incidence. Standard regression assumptions, including normality of residuals and homoscedasticity, were verified for all final models. Multicollinearity was assessed using variance inflation factors (VIF). All statistical analyses were performed using R software, and a *p*-value < 0.05 was considered statistically significant.

## 3 Results

### 3.1 Annual trends and sex differences

**Figure 1:**
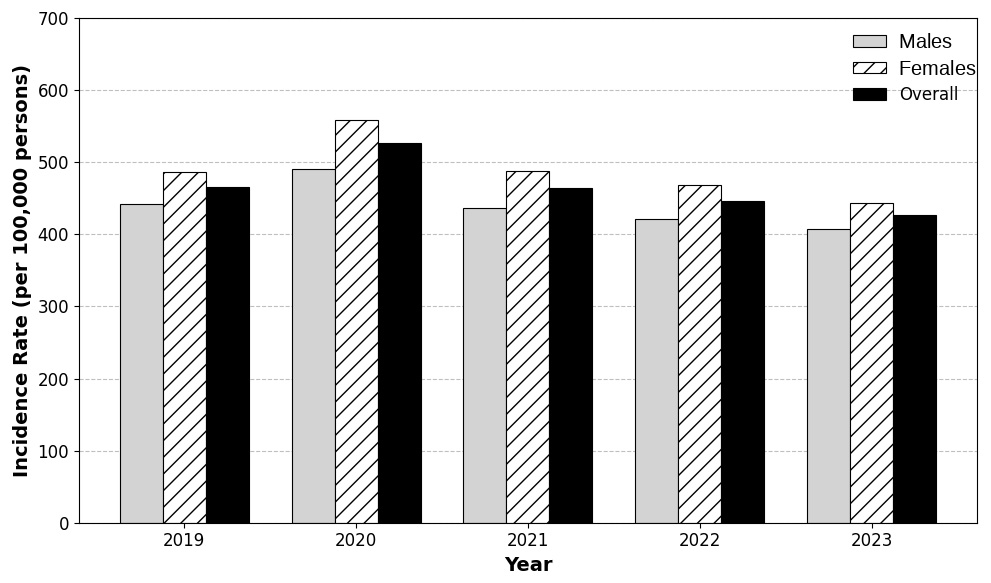
Incidence of irritable bowel syndrome in the overall population and by sex from 2019 to 2023. The incidence showed a declining trend after 2020, with females consistently exhibiting higher rates than males throughout the study period.

Changes in the prevalence and incidence rate by year based on CDM data are shown in Table 1. The prevalence of IBS in the overall population and in males and females increased until 2021, followed by a decline thereafter. The prevalence rate was consistently higher in females than in males for all years. Comparing 2019 and 2023, the overall prevalence rate slightly increased from 68.38 to 69.64 per 100,000 persons. Specifically, the rate for males decreased from 65.01 to 62.13 per 100,000 persons, whereas the rate for females increased from 71.34 to 76.02 per 100,000 persons. Conversely, the overall incidence rate of IBS has shown a decreasing trend since 2020 (Table 1 and Figure 1). Between 2019 and 2023, the overall incidence rate dropped from 465.29 to 427.25 per 100,000 persons. This decline was observed in both sexes; incidence in males decreased from 441.44 to 407.77 per 100,000 persons, and in females from 486.20 to 443.79 per 100,000 persons. Notably, the sex gap in the incidence rate has progressively narrowed each year since 2020.

**Table 1:** Prevalence and incidence of irritable bowel syndrome in the overall population and by sex (unit: per 100,000 persons).

Year	Overall		Males		Females	
	*n*	Rate	*n*	Rate	*n*	Rate
Prevalence (per 100,000 persons)						
2019	858	68.38	381	65.01	477	71.34
2020	912	72.92	402	68.77	510	76.57
2021	1,523	114.39	653	106.97	870	120.67
2022	1,164	85.00	479	76.36	685	92.30
2023	979	69.64	401	62.13	578	76.02
Incidence (per 100,000 persons)						
2019	5,838	465.29	2,587	441.44	3,251	486.20
2020	6,590	526.94	2,867	490.47	3,723	558.96
2021	6,186	464.61	2,668	437.03	3,518	487.97
2022	6,117	446.68	2,639	420.70	3,478	468.63
2023	6,006	427.25	2,632	407.77	3,374	443.79
Total	36,173		15,709		20,464	

### 3.2 Age-specific patterns

**Figure 2:**
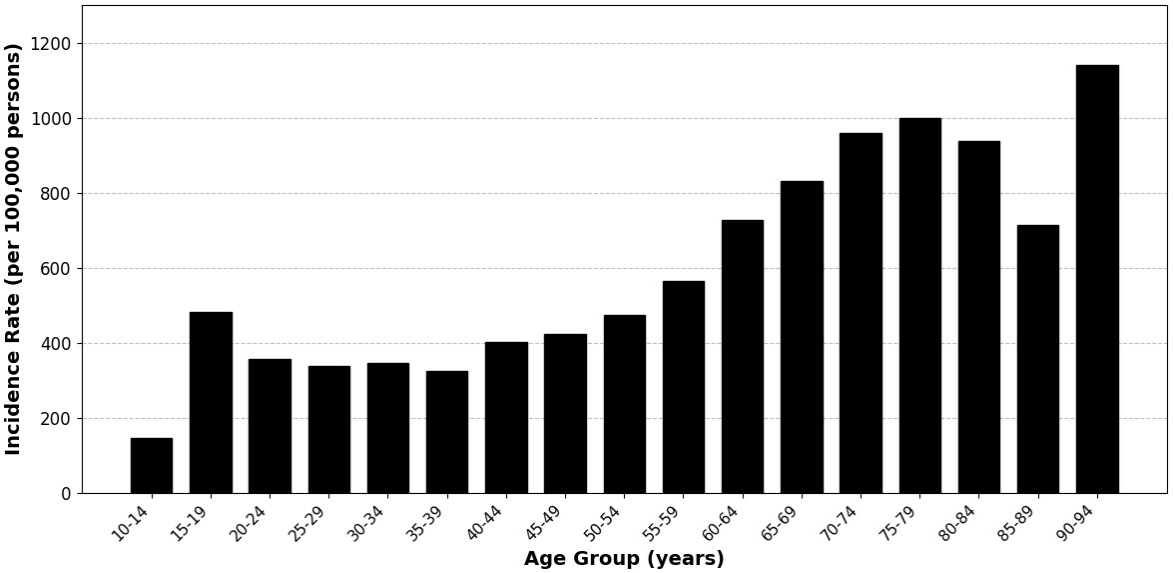
Incidence of irritable bowel syndrome by age group from 2019 to 2023. Incidence rates increased with age, peaking in the older population (≥65 years), with a notable high incidence also observed in adolescents (15–19 years).

The prevalence and incidence rates by age group continued to increase up to the 70–79-year age group, with the exception of a high incidence spike in the 15–19-year age group (Table 2 and Figure 2). This distinct pattern remained consistent from 2019 to 2023.

**Table 2:** Prevalence (2023) and 5-year average incidence (2019–2023) of irritable bowel syndrome by age group (unit: per 100,000 persons).

Age range	Prevalence (unit: per 100,000 persons)	Incidence (unit: per 100,000 persons)
10–14 years	6.15	144.58
15–19 years	25.65	480.40
20–24 years	9.96	357.49
25–29 years	22.18	337.55
30–34 years	51.33	345.97
35–39 years	76.32	325.25
40–44 years	103.64	402.68
45–49 years	119.41	423.15
50–54 years	117.56	473.66
55–59 years	89.86	563.78
60–64 years	122.88	726.37
65–69 years	124.98	830.58
70–74 years	128.39	959.16
75–79 years	64.98	998.32
80–84 years	36.50	936.50
85–89 years	44.30	712.90
90–94 years	39.32	1,139.60

### 3.3 Seasonal variation and meteorological associations

**Figure 3:**
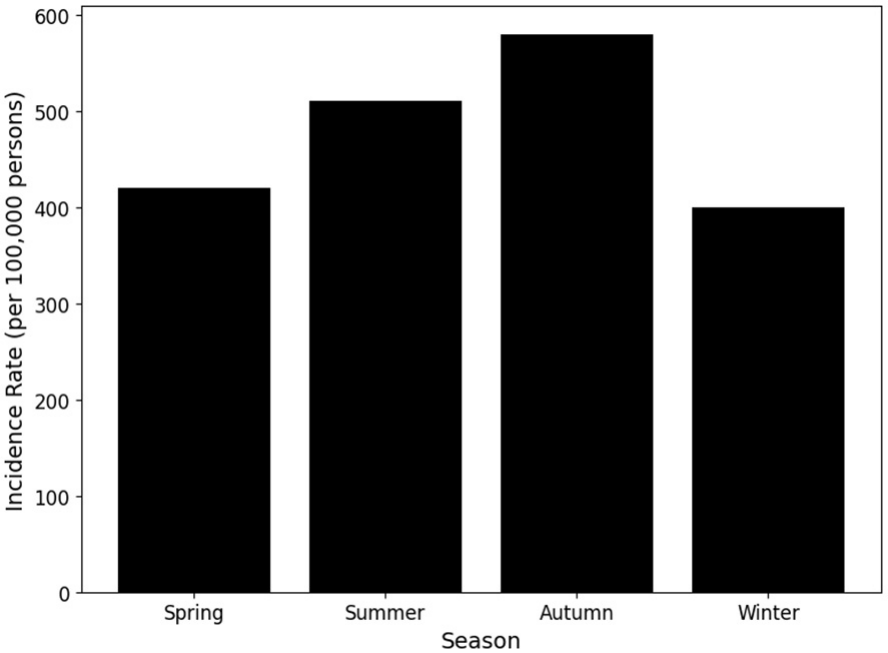
Incidence of irritable bowel syndrome by season from 2019 to 2023. A clear seasonal variation was observed, with the highest incidence occurring in autumn, followed by summer, and the lowest in winter.

**Figure 4:**
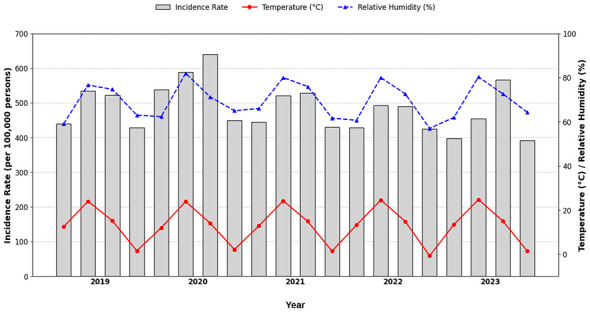
Seasonal variation in irritable bowel syndrome incidence, average temperature, and average relative humidity from 2019 to 2023. The bars represent the quarterly incidence rate of IBS, displayed in chronological order (Spring, Summer, Autumn, and Winter) for each year. The solid line indicates average temperature, and the dashed line indicates average relative humidity. Fluctuations in incidence closely paralleled changes in temperature and humidity, demonstrating a positive correlation with environmental factors.

The seasonal incidence of IBS was highest in autumn, followed by summer, and lowest in spring and winter (Figure 3). During the study period, the average temperature ranged from 1.13 °C to 24.25 °C, and the average relative humidity ranged from 61.4% to 79.8% (Figure 4). The multivariable regression models, adjusted for the calendar year, showed that the mean seasonal relative humidity had a statistically significant association with the incidence rate of IBS [β = 4.97, 95% CI [2.11, 7.83]]. This effect size implies that a 10-percentage-point increase in relative humidity is associated with approximately 50 additional IBS cases per 100,000 persons. Additionally, a significant positive association was noted between the mean seasonal temperature and the incidence rate of IBS [β = 4.36, 95% CI [1.46, 7.26]], indicating an absolute increase of 4.36 incident cases per 100,000 persons for every 1 °C increase in temperature (Table 3). In the lag effect analysis, humidity from the preceding season was not significantly associated with IBS incidence, suggesting that the environmental impact is likely acute rather than delayed. Diagnostic tests confirmed that the final models met all statistical assumptions, with all VIF values below 1.1, indicating negligible multicollinearity.

**Table 3:** Regression analysis of the average relative humidity and temperature with the incidence rate of irritable bowel syndrome.

Meteorological factors	Univariate			Multivariable		
	Coefficient (β)	95% CI	*p*-value	Coefficient (β)	95% CI	*p*-value
Humidity	5.12	(1.86, 8.37)	0.004	4.97	(2.11, 7.83)	0.002
Temperature	4.29	(0.95, 7.63)	0.015	4.36	(1.46, 7.26)	0.006

CI, confidence interval.

β represents the absolute change in the seasonal IBS incidence rate per 100,000 persons associated with a 1-percentage-point increase in mean relative humidity or a 1 °C increase in mean temperature. In the multivariable models, each meteorological factor was analyzed in a separate model adjusted for calendar year.

### 3.4 Validation with nationwide HIRA data

**Figure 5:**
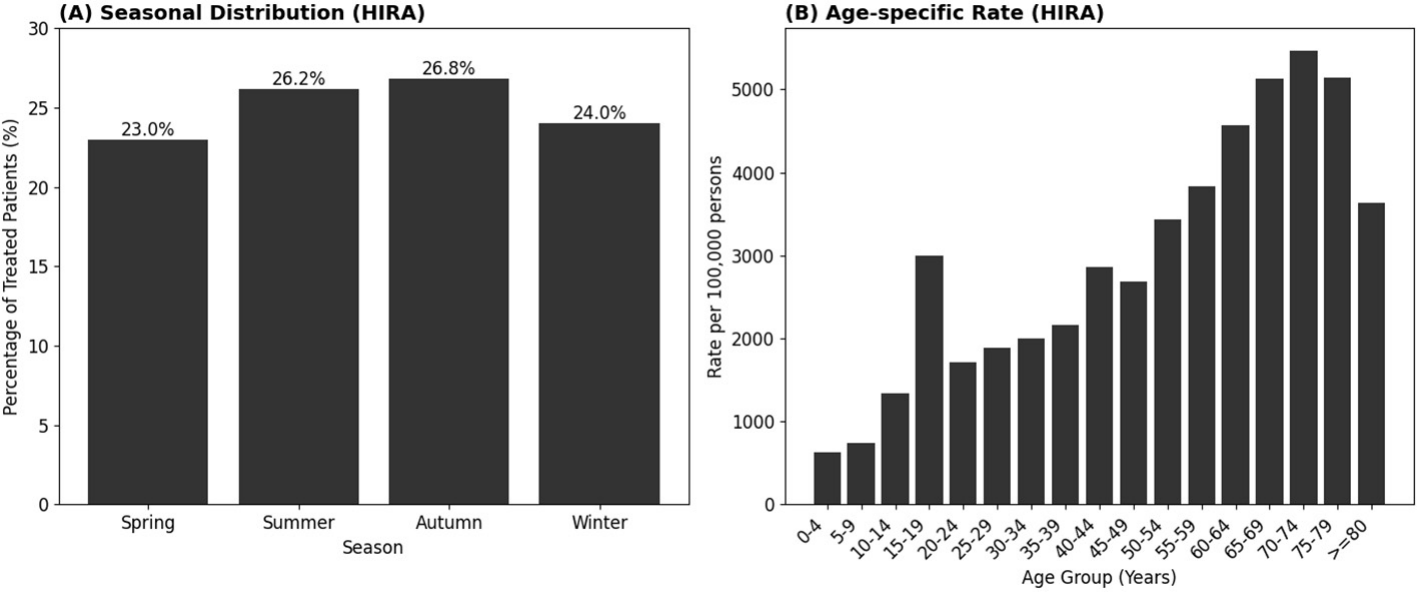
Validation using nationwide data. Comparison of IBS epidemiological patterns using the Health Insurance Review and Assessment (HIRA) database (2019–2023). **(A)** Seasonal distribution of patients treated for IBS, showing peaks in autumn and summer. **(B)** Age-specific rate of patients treated for IBS, illustrating an increasing trend with age. HIRA data represents the rate of patients who utilized medical services (period prevalence), serving to validate the epidemiological trends observed in the CDM cohort.

To validate the representativeness of our multicenter CDM findings, we performed a parallel analysis using the nationwide Health Insurance Review and Assessment (HIRA) database, which covers approximately 96% of the Korean population (12, 13). Although the HIRA data represents the overall proportion of patients treated for IBS (period prevalence)—differing from the strict incidence definition used in our CDM analysis—the epidemiological patterns were remarkably consistent (Figure 5).

Specifically, the seasonal variation observed in the HIRA data (Figure 5A) mirrored the trends in our CDM cohort, showing distinct peaks in autumn and summer. Furthermore, the age-specific analysis (Figure 5B) confirmed the increasing burden of IBS in the older population, consistent with our primary findings. Detailed annual trends and raw data for the nationwide population are provided in Supplementary Figure S1 and Supplementary Tables S1, S2.

## 4 Discussion

### 4.1 Temporal trends and diagnostic criteria

Our study demonstrates a low prevalence (< 1%) and a declining incidence of IBS in Korea between 2019 and 2023. These rates are substantially lower than the global prevalence of 3.8–11.0% reported in previous studies using various criteria (2, 4, 14–20). The relatively low figures observed in our study may be partially explained by the adoption of the stricter Rome IV criteria in recent years, which requires at least weekly abdominal pain, compared to the less stringent Rome III criteria (1, 21). A summary of recent studies reporting the prevalence and incidence of IBS based on the Rome IV criteria is presented in Table 4 (15–20). Consequently, it is plausible that patients previously diagnosed with IBS might now be classified with other functional bowel disorders, such as functional constipation or diarrhea, although further clinical validation is required to confirm this shift in real-world practice (22–25). Furthermore, the discrepancy between the immediate decline in incidence after 2020 and the delayed decline in prevalence after 2021 reflects the chronic nature of IBS, where the reduction in new diagnoses precedes the decline in the cumulative number of existing cases. These declining trends and low prevalence align with recent findings from Taiwan and Japan, suggesting regional specificities in East Asia (26, 27).

**Table 4:** Reported irritable bowel syndrome prevalence in studies conducted in the past 5 years.

Study	Location	Study period	Data collection method	Criteria	Population size (unit: person)	Prevalence (%)	Sex ratio (F/M)
Sokpon et al. (15)	Africa (Cotonou)	2021	Questionnaire	Rome IV	768	4.2	1.67
Almario et al. (16)	USA	2020	Questionnaire	Rome IV	88,607	6.1	NR
Galica et al. (17)	Albania	2019–2020	Questionnaire	Rome IV	502	8.6	≈1.0
Kovács et al. (18)	Gibraltar	2019–2020	Questionnaire	Rome IV	888	5.2	NR
Abdel-Qader et al. (19)	Jordanian	2023	Online Questionnaire^β^	Rome IV	1,042	41.7	3.06
Guido et al. (20)	Uruguay	2021	Online Questionnaire^γ^	Rome IV	1,052	17.1	9.85

NR, Not Reported (Data required to calculate the exact sex ratio within the IBS group was not available in the cited reference).

^β^The study by Abdel-Qader et al. (19) utilized online convenience sampling via social media, likely resulting in an overestimation of prevalence due to selection bias.

^γ^The study by Guido et al. (20) utilized online convenience sampling. The exceptionally high sex ratio is attributed to a significant gender imbalance among participants (approximately 79% female).

### 4.2 Sex and age differences

Consistent with global trends, our study observed higher IBS rates in females than in males (28). This female predominance is frequently attributed to sex-specific differences in stress responses, pain modulation, and the influence of sex hormones on gastrointestinal motility (29–32). However, the sex gap in our study was relatively narrow and has been progressively decreasing since 2020. This narrowing gap, often observed in Asian cohorts compared to Western populations, could be influenced by evolving healthcare-seeking behaviors among men and region-specific sociocultural factors (33, 34). Regarding age, we observed a dual-peak pattern: a continuously increasing burden among older adults (≥65 years) and a notably high incidence in adolescents (15–19 years). The former likely reflects Korea's rapidly aging demographic (35), while the latter may be associated with significant psychological distress, such as severe academic stress, which is known to impact the brain-gut axis in adolescents (36).

### 4.3 Seasonal variation and environmental factors

Beyond demographic factors, a key finding of our study is the significant seasonal variation in IBS incidence, which peaked in autumn and summer and showed positive correlations with ambient temperature and humidity. In autumn, the increased prevalence of allergic and respiratory illnesses may influence immune function and potentially exacerbate intestinal symptoms (37, 38). The summer peak may be multifactorial: hot and humid conditions foster foodborne pathogens, potentially increasing the risk of post-infectious IBS (39), while temperature stress from frequent exposure to air conditioning and cold foods can alter visceral sensitivity and gut microbiota balance (40–44). These associations highlight the potential role of environmental factors in IBS exacerbation.

### 4.4 Limitations

This study has several limitations. First, the identification of IBS relied on ICD-10 diagnostic codes (K58) mapped to the CDM, rather than direct clinical symptom questionnaires. This introduces potential coding bias, as physicians in real-world practice may not strictly adhere to the Rome IV criteria when assigning billing codes, which could affect the precise estimation of incidence and prevalence. Second, as our data were derived from tertiary hospitals, there is an inherent healthcare-seeking bias. Our cohort captures only patients who actively sought specialized medical care, likely underrepresenting individuals with milder symptoms who self-manage their condition without visiting specialized medical institutions. Third, the study lacked detailed clinical information, such as symptom severity, dietary habits, and psychiatric comorbidities. Despite these limitations, the consistent epidemiological patterns observed in our multicenter CDM cohort—and validated against the nationwide HIRA database—support the robust external validity of our findings regarding seasonal and demographic trends.

## 5 Conclusion

In conclusion, both the prevalence and incidence of IBS in Korea have decreased between 2019 and 2023. These trends may partially reflect the adoption of the stricter Rome IV diagnostic criteria. Females consistently exhibited higher rates than males, although this sex gap has progressively narrowed over time. Furthermore, the highest incidence was observed among older adults and adolescents. Significant seasonal variation was also evident, with incidence peaking in autumn and summer, which positively correlated with ambient temperature and humidity. These findings highlight the need to consider sex- and age-specific disparities, along with environmental determinants, in understanding IBS epidemiology. Future large-scale, prospective studies are warranted to validate these associations and to inform targeted, population-based management strategies aimed at reducing the public health burden of IBS.

## Data Availability

The original contributions presented in the study are included in the article/Supplementary material, further inquiries can be directed to the corresponding author.
