# Supplementary material for: Trends and seasonal variation in the incidence and prevalence of irritable bowel syndrome in Korea: a multicenter OMOP CDM study
Source: Front Public Health. 2026 Jun 5;14:1711919. doi: 10.3389/fpubh.2026.1711919 (PMC13279324; doi:10.3389/fpubh.2026.1711919)
Supplement: Supplementary file 1 [file Data_Sheet_1.pdf]

Supplementary Material

1 Supplementary Figures and Tables

1.1 Supplementary Figures

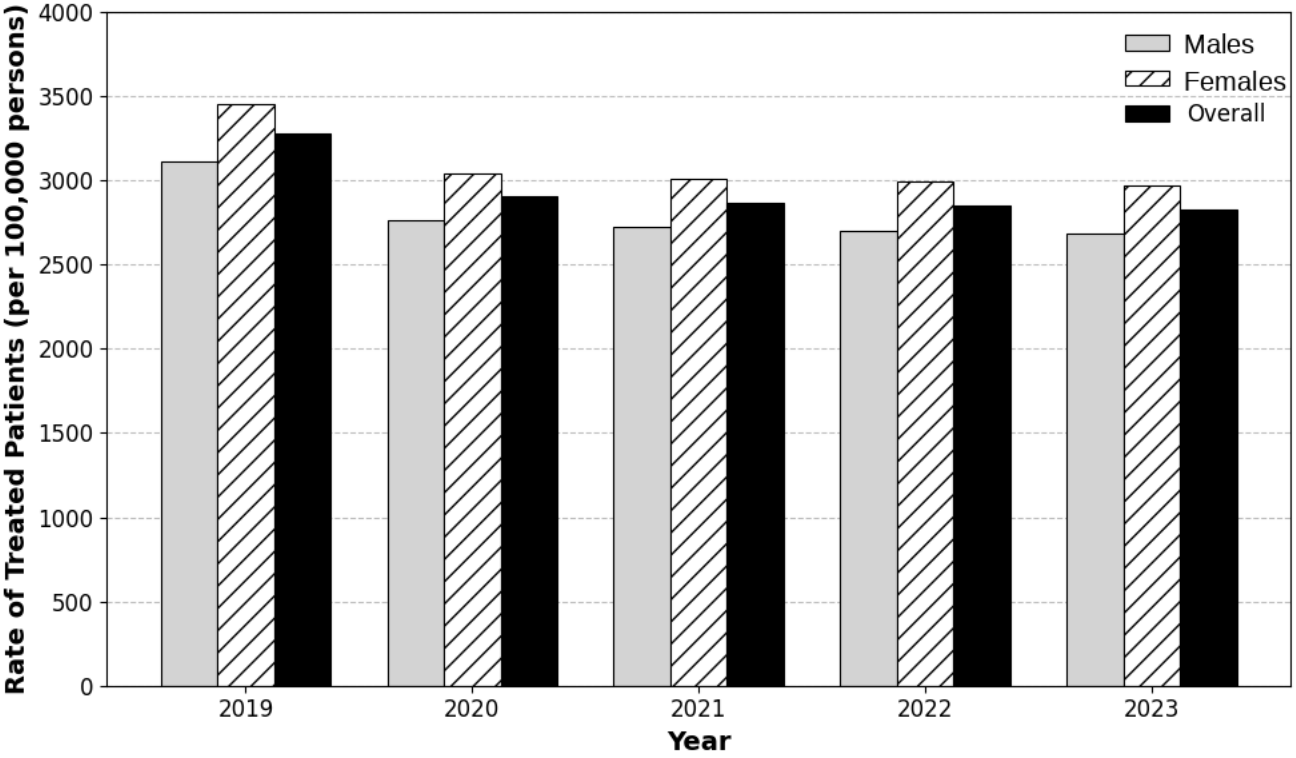

Figure S1. The rate (per 100,000 persons) of patients treated for irritable bowel syndrome by overall and sex from 2019 to 2023 extracted from the Health Insurance Review and Assessment (HIRA) in Korea.

1.2 Supplementary Tables

Table S1. The number and rate (per 100,000 persons) of patients treated for irritable bowel syndrome extracted from the Health Insurance Review and Assessment (HIRA) in Korea.

Year	Overall		Males		Females	
	n	rate	n	rate	N	rate
2019	1,696,615	3,282	803,783	3,109	892,832	3,454
2020	1,501,235	2,904	714,364	2,763	786,871	3,044
2021	1,480,929	2,864	703,980	2,723	776,949	3,006
2022	1,471,829	2,847	697,749	2,699	774,080	2,995
2023	1,462,523	2,829	694,874	2,688	767,649	2,970

Table S2. The number and rate (per 100,000 persons) of patients treated for irritable bowel syndrome by age group from 2019 to 2023 extracted from the Health Insurance Review and Assessment (HIRA) in Korea.

Year / Age range	2019		2020		2021		2022		2023	
	n	rate	n	rate	n	rate	n	rate	n	rate
**0-4 years**	20,117	1,042	10,787	607	7,607	470	8,300	556	6,449	465
**5-9 years**	26,354	1,158	15,970	701	12,184	546	13,098	611	13,993	690
**10-14 years**	40,737	1,817	27,669	1,229	24,978	1,087	30,221	1,307	28,788	1,257
**15-19 years**	90,717	3,377	66,936	2,658	65,264	2,782	75,820	3,314	66,324	2,882
**20-24 years**	72,139	2,098	58,431	1,722	52,852	1,612	50,339	1,621	45,551	1,542
**25-29 years**	81,713	2,278	72,100	1,978	66,053	1,797	62,447	1,721	59,881	1,666
**30-34 years**	74,794	2,262	67,275	2,034	64,319	1,945	64,530	1,903	66,581	1,889
**35-39 years**	96,449	2,380	82,813	2,142	78,267	2,125	72,241	2,074	70,076	2,092
**40-44 years**	117,730	3,060	108,750	2,809	111,659	2,831	111,981	2,775	115,070	2,830
**45-49 years**	130,270	2,903	113,104	2,579	110,475	2,622	106,389	2,619	105,948	2,698
**50-54 years**	164,313	3,823	147,443	3,399	148,345	3,352	148,283	3,303	149,292	3,299
**55-59 years**	188,654	4,392	165,398	3,904	154,145	3,747	147,977	3,597	143,825	3,533
**60-64 years**	187,050	5,156	175,800	4,609	182,505	4,490	180,515	4,352	179,211	4,209
**65-69 years**	140,927	5,755	137,940	5,181	146,196	5,050	149,164	4,869	156,852	4,791
**70-74 years**	115,695	6,134	109,372	5,495	113,406	5,425	113,162	5,227	112,611	5,039
**75-79 years**	91,234	5,739	84,422	5,249	80,482	5,135	77,594	4,860	77,558	4,738
**≥80 years**	73,415	4,160	70,847	3,747	71,806	3,560	72,861	3,379	76,740	3,351
**Total**	1,712,308		1,515,057		1,490,543		1,484,922		1,474,750	
